# Occurrence of Multidrug-Resistant Bacteria Resulting from the Selective Pressure of Antibiotics: A Comprehensive Analysis of ESBL *K. pneumoniae* and MRSP Isolated in a Dog with Rhinorrhea

**DOI:** 10.3390/vetsci10050326

**Published:** 2023-05-02

**Authors:** Inês C. Rodrigues, Marisa Ribeiro-Almeida, Jorge Ribeiro, Leonor Silveira, Joana C. Prata, Angela Pista, Paulo Martins da Costa

**Affiliations:** 1 School of Medicine and Biomedical Sciences, University of Porto (ICBAS-UP), Rua de Jorge Viterbo Ferreira, 228, 4050-313 Porto, Portugal; icrodrigues@icbas.up.pt (I.C.R.); up201008465@edu.icbas.up.pt (M.R.-A.); jribeiro@icbas.up.pt (J.R.); joana.prata@iucs.cespu.pt (J.C.P.); 2 Interdisciplinary Centre of Marine and Environmental Research (CIIMAR), Terminal de Cruzeiros do Porto, de Leixões, Av. General Norton de Matos s/n, 4450-208 Matosinhos, Portugal; 3 UCIBIO—Applied Molecular Biosciences Unit, Rede de Química e Tecnologia (REQUIMTE), Laboratory of Microbiology, Department of Biological Sciences, Faculty of Pharmacy, University of Porto, 4050-313 Porto, Portugal; 4 Department of Infectious Diseases, National Institute of Health, Av. Padre Cruz, 1600-609 Lisbon, Portugal; leonor.silveira@insa.min-saude.pt (L.S.); angela.pista@insa.min-saude.pt (A.P.); 5 TOXRUN—Toxicology Research Unit, University Institute of Health Sciences, Cooperativa de Ensino Superior Politécnico e Universitário (CESPU), 4585-116 Gandra, Portugal

**Keywords:** antibiotic pressure, dog, multidrug-resistant bacteria, ESBL *Klebsiella pneumoniae*, MRSP *Staphylococcus pseudintermedius*

## Abstract

**Simple Summary:**

Antimicrobial resistance (AMR) poses a major threat to human and animal health. One of the causes underlying the emergence of increasingly resistant strains is antibiotic selective pressure. This study aimed to evaluate the impact of treatment with amikacin on an extended spectrum β-lactamase (ESBL)-producing *Klebsiella pneumoniae* isolated in a dog with rhinorrhea. In the middle of the treatment, methicillin-resistant *Staphylococcus pseudintermedius* (MRSP) was isolated from the left nasal cavity of the dog. At the end of the treatment, *K. pneumoniae* was not recovered from nasal swab samples, while MRSP displayed phenotypical and genotypical changes. Six weeks after the end of the treatment, only commensal flora was observed in both nasal cavities. These results warn of the effects of antibiotic pressure, which can lead to the emergence of multidrug-resistant strains either by directly promoting the enrichment of bacteria with resistance to multiple antimicrobial agents or via the subsequent acquisition of resistance genes. Therefore, adapting clinical practice to this new reality is crucial to limit the selection and spread of multi-resistant bacteria among pets, humans and the environment.

**Abstract:**

Because of public health concerns, much greater scrutiny is now placed on antibiotic use in pets, especially for antimicrobial agents that have human analogs. Therefore, this study aimed to characterize the phenotypic and genotypic profiles of multidrug-resistant bacteria isolated from nasal swabs samples taken from a one-year-old male Serra da Estrela dog with rhinorrhea that was treated with amikacin. An extended-spectrum β-lactamases (ESBL) *Klebsiella pneumoniae* was isolated in the first sample taken from the left nasal cavity of the dog. Seven days later, methicillin-resistant (MRSP) *Staphylococcus pseudintermedius* was also isolated. Nevertheless, no alterations to the therapeutic protocol were performed. Once the inhibitory action of the antibiotic disappeared, the competitive advantage of the amikacin-resistant MRSP was lost, and only commensal flora was observed on both nasal cavities. The genotypic profile of extended-spectrum β-lactamase (ESBL)-producing *Klebsiella pneumoniae* revealed the same characteristics and close relation to other strains, mainly from Estonia, Slovakia and Romania. Regarding MRSP isolates, although resistance to aminoglycosides was present in the first MRSP, the second isolate carried *aac(6′)-aph(2″)*, which enhanced its resistance to amikacin. However, the veterinary action was focused on the treatment of the primary agent (ESBL *K. pneumoniae*), and the antibiotic applied was according to its phenotypic profile, which may have led to the resolution of the infectious process. Therefore, this study highlights the importance of targeted therapy, proper clinical practice and laboratory-hospital communication to safeguard animal, human and environmental health.

## 1. Introduction

Antimicrobial resistance (AMR) poses a major threat to human and animal health worldwide [1,2]. The indiscriminate use and overuse of antimicrobials are some of the most important causes underlying the emergence of increasingly resistant strains through selective pressure [3,4,5]. Empirical antibiotic therapy frequently uses broad-spectrum antimicrobials or combinations of antimicrobials, which may also be required in polymicrobial infections or life-threatening conditions [6]. Indeed, 76% of veterinary clinicians support antimicrobial selection based on personal experience [4]. In addition to veterinary and human medicines sharing antibiotics, the transmission of resistant bacteria may occur due to the proximity between humans and companion animals [7,8].

Antibiotic therapy, either empirical or pathogen-directed, exerts selective pressure triggering multiple survival strategies resulting in vertical (mutations) or horizontal (of mobile genetic elements) transmission [9]. Bacterial populations present a notorious adaptative potential and high plasticity when facing different types of stress, including antibiotic therapy [9,10]. Indeed, multidrug-resistant strains can be selected under antibiotic pressure as a result of antibiotic treatment [11,12]. Therefore, physicians and veterinarians are important actors in the control of antimicrobial resistance as part of “One Health,” especially for critical pathogens (WHO, ECDC) [13,14,15,16].

*Klebsiella pneumoniae* (*K. pneumoniae*) is one of the leading pathogens associated with the emergence of antibiotic resistance and a clinically significant nosocomial pathogen, also associated with high morbidity and mortality rates in companion animals [17,18]. Another emerging zoonotic pathogen of canine origin is methicillin-resistant *Staphylococcus pseudintermedius* (*S. pseudintermedius*; MRSP), which is transmitted by direct contact with or bites to pet owners or veterinary staff [19].

This study aimed at characterizing the phenotypic and genotypic profiles of multidrug-resistant strains isolated during a single infectious process, evaluating the impact of antibiotic selective pressure. A single case study of a dog presenting with mucopurulent rhinorrhea caused by an extended spectrum β-lactamases (ESBL) producing *K. pneumoniae* was investigated, followed by the appearance of MRSP. Antimicrobial resistance profiling and Whole Genome Sequencing (WGS) were performed on the strains isolated from this case. The impact of the applied antibiotic, amikacin, was evaluated during the therapeutic protocol on the nasal flora of the animal.

## 2. Materials and Methods

### 2.1. Case Selection

Cases admitted to the Veterinary Hospital (UPVET) of the Institute of Biomedical Sciences Abel Salazar, University of Oporto (ICBAS/UP) from the 1st of January 2022 to the 31st of December 2022 were analyzed (n = 8338). Eligibility criteria for case enrolment were: (i) admission to the UPVET for bacteriological infection, (ii) follow-up performed by UPVET, (iii) sending of more than one consecutive sample to the microbiology laboratory of ICBAS-UP during the same infectious process, (iv) isolation of a pure bacterial culture with a multidrug-resistant profile and clinically relevant under the One Health approach. A single case was selected based on these criteria. Informed consent was obtained from all of the UPVET clients for the use of data of patients for scientific study and teaching purposes. Data was safely stored and anonymized according to Data Protection laws (Regulation (EU) 2016/679).

**Figure 1:**
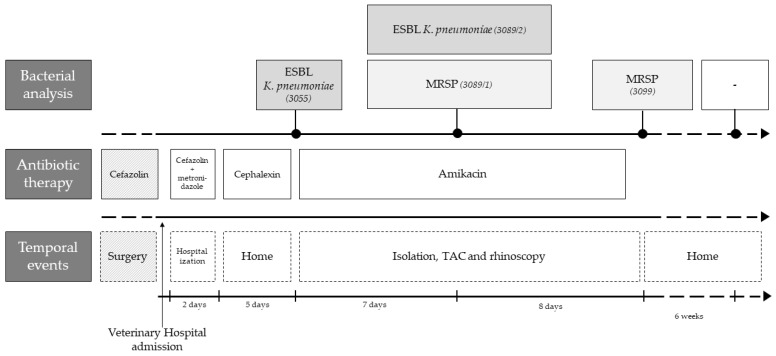
Timeline of the clinical case highlighting the main temporal events, antibiotic therapy and bacterial analysis.

The selected case pertains to a 1-year-old, unneutered, giant breed (Serra da Estrela) dog with up-to-date vaccination and deworming, followed at UPVET. The owner of this animal had requested an emergency appointment since his dog showed signs of vomiting and mucopurulent rhinorrhea from the left nostril (Figure 1). Clinical examination revealed pain on abdominal palpation and mucopurulent rhinorrhea from the left nostril with no other abnormalities. The dog had been submitted for the surgical correction of a gastric dilatation volvulus a week earlier. Therefore, the clinicians decided to hospitalize the animal until the vomiting stopped. During hospitalization, supportive medication was administered, keeping the antibiotic treatment instituted by the hospital where the surgery was performed, consisting of cephazolin and metronidazole. After 2 days, the dog returned home with gastric support medication, along with a cephalexin prescription. After the first microbiological result of the left nasal cavity, the dog was again hospitalized, and a computed tomography (CT) scan was performed to understand the severity of the infectious process in the nasal cavities. The CT scan showed rhinosinusitis in the left nasal cavity, decreased nasal turbinates in the middle cavity and homogeneous material partially occupying the left nasal cavity (Appendix A). In order to discard fungal involvement, a rhinoscopy was performed, in which no signs of fungal plaques were detected. Besides, the biochemical analysis of blood to monitor urea and creatinine was also performed, and no alterations were recorded. In parallel, 4 samples were collected in order to follow the microbiological evolution of the clinical case. Samples were collected at 3 different times during antibiotic treatment: 8 and 18 days after the antibiotics’ start and 6 weeks after the end of antibiotic treatment.

### 2.2. Sample Collection and Bacterial Isolation

Samples were collected using a sterile swab and vigorously rubbing the most caudal side of the nasal cavity, as previously described by the Centers for Disease Control and Prevention (CDC) [20]. The collected samples were immediately transported to the microbiology laboratory and processed within 2 h of collection.

**Figure 2:**
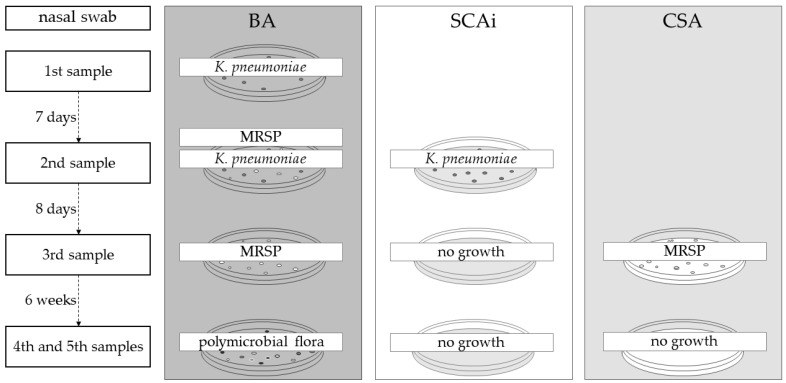
Culture media used in the microbiological analysis of the five samples. Additional culture media were added to subsequent samples to specifically culture the previously identified bacteria. BA: Blood agar, SCAi: Simmons Citrate agar contain 1% of inositol, CSA: CHROMagar™ *S. aureus* (CSA).

The first analysis was processed according to UK Standards for Microbiology Investigations [21]. As ESBL *K. pneumoniae* was isolated in the first sample, Simmons Citrate Agar (SCA) containing 1% (*w*/*v*) of myo-inositol (SCAi) was used along with blood agar media (BA, Tryptone Soy Agar containing 5% of laked horse blood Agar). In the third sample, CHROMagar™ *Staphylococcus aureus* (CSA) was also used since, in the second sample, *K. pneumoniae* and MRSP were isolated. Finally, in the fourth sample, these 3 culture mediums were used: BA, CSA and SCAi. The schematic representation of the culture media used throughout the five samples is shown in Figure 2.

The plates with BA and CSA were incubated for 24 h, while the SCAi plates were left for 48 h at 37 °C. Bacterial isolates obtained from BA were Gram-stained and identified with conventional biochemical tests. Mauve to purple colonies growing on CSA were suspected to be coagulase-positive Staphylococcus. Moreover, yellow, dome-shaped, often mucoid colonies growing on SCAi were suspicious for *Klebsiella* spp. To confirm at the species level of *Klebsiella* spp. isolates, the RapID™ ONE System (Thermo Fischer Scientifics, Waltham, MA, USA) was used.

All isolated colonies were frozen in buffered peptone water (BPW) containing 1.5% (*v*/*v*) glycerol at −20 °C.

### 2.3. Antimicrobial Susceptibility Testing

Antimicrobial susceptibility testing was performed and interpreted according to the Clinical and Laboratory Standards Institute guidelines [22] using the Kirby–Bauer method. Antimicrobials were selected to represent a wide range of classes, and selection between different antimicrobial agents of the same class was based on the availability of clinical CLSI breakpoints [23].

A panel of 19 antimicrobials was used for *K. pneumoniae*: amikacin (AMK, 30 μg), amoxicillin/clavulanic acid (AMC, 30 μg), ampicillin (AMP, 10 μg), azithromycin (AZM, 15 μg), aztreonam (ATM; 30 μg), cefotaxime (CTX; 30 μg), cefoxitin (FOX; 30 μg), ceftazidime (CAZ; 30 μg), cephazolin (CFZ; 30 μg), chloramphenicol (CHL; 30 μg), ciprofloxacin (CIP; 5 μg), doxycycline (DOX; 30 μg), gentamycin (GEN; 120 μg), imipenem (IMP; 10 μg), levofloxacin (LEV; 5 μg), nitrofurantoin (NIT; 300 μg), streptomycin (STR; 10 μg), sulfamethoxazole/trimethoprim (SXT; 25 μg), tetracycline (TET; 30 μg), and tobramycin (TOB; 10 μg). For *S. pseudintermedius,* 17 antimicrobials were tested: azithromycin (AZM; 15 μg), cefoxitin (FOX; 30 μg), chloramphenicol (CHL; 30 μg), ciprofloxacin (CIP; 5 μg), clindamycin (CLI; 2 μg), doxycycline (DOX; 30 μg), erythromycin (ERY; 15 μg), gentamycin (GEN; 120 μg), levofloxacin (LEV; 5 μg), linezolid (LZD; 30 μg), nitrofurantoin (NIT; 300 μg), oxacillin (OXA; 1 μg), penicillin (PEN; 10 UI), quinupristin-dalfopristin (QDA; 15 μg), rifampicin (RIF; 5 μg), sulfamethoxazole/trimethoprim (SXT; 25 μg), tetracycline (TET; 30 μg), and tobramycin (TOB; 10 μg). All antimicrobial disks were from Oxoid (Basingstoke, UK).

Bacterial isolates were classified as susceptible, intermediate or resistant using current CLSI breakpoints [23]. Isolates resistant to 3 or more antibiotics classes were defined as multidrug-resistant (MDR) bacteria [24].

### 2.4. DNA Extraction and WGS Tecnhique

Genomic DNA was extracted from fresh cultures of each isolate using the Isolate II Genomic DNA Kit (Bioline, London, UK), followed by quantification in the Qubit fluorometer (Invitrogen, Waltham, MA, USA) with the dsDNA HS Assay Kit (Thermo Fisher Scientific, Waltham, MA, USA), according to the manufacturer’s instructions. The DNA was subjected to the NexteraXT library preparation protocol (Illumina, San Diego, CA, USA) prior to cluster generation and paired-end sequencing (2 × 150 bp) on a NextSeq 550 instrument (Illumina), according to the manufacturer’s instructions. FastQC v0.11.5 (https://www.bioinformatics.babraham.ac.uk/projects/fastqc/ (accessed on 16 February 2023)) was used for quality control and Trimmomatic v0.38 [25] for trimming low-quality bases.

#### 2.4.1. Bioinformatic Analysis of *K. pneumoniae*

Online bioinformatic tools from PathogenWatch v20.0.13 (https://pathogen.watch/; accessed on 8 February 2023), specifically, Kleborate v2.2.0, were used to evaluate *K. pneumoniae* antibiotic resistance genes or known mutations, virulence genes, plasmid typing, Multilocus Sequence Typing (MLST) [26], core genome Multilocus Sequence Typing (cgMLST), capsular polysaccharide (K) and lipopolysaccharide O locus types and serotypes [27]. The phylogenetic analysis inferred by the neighbor-joining tree was based on the Pathogenwatch pairwise-distance matrix, based on the single nucleotide polymorphism (SNP) distances of a core gene library (1972 genes) [28]. Closely related genomes and the associated metadata (country, source and date) were collected from all public genomes available from Pathogenwatch after cgMLST single-linkage clustering and the selection of those with less than 5 allele differences. The neighbor-joining tree was edited using iToL [29].

#### 2.4.2. Bioinformatic Analysis of *S. pseudintermedius*

For bioinformatic analysis for *S. pseudintermedius* strains, DNA was assembled using the Bacterial and Viral Bioinformatics Resource Center (BV-BRC) platform (https://www.bv-brc.org/app/Assembly2; accessed on 8 February 2023). Moreover, tools from Centre for Genomic and Epidemiology (http://www.genomicepidemiology.org; accessed on 26 January 2023) were used to assess antibiotic resistance genes or known mutations (ResFinder 4.1; https://cge.food.dtu.dk/services/ResFinder/; accessed on 9 February 2023), virulence genes (VirulenceFinder 2.0; https://cge.food.dtu.dk/services/VirulenceFinder/; accessed on 9 February 2023), plasmid replicons (PlasmidFinder 2.1; https://cge.food.dtu.dk/services/PlasmidFinder/; accessed on 16 February 2023), SCC*mec* elements (SCCmecFinder 1.2; https://cge.food.dtu.dk/services/SCCmecFinder/; accessed on 16 February 2023) and Multilocus Sequence Typing (MLST 2.0; https://cge.food.dtu.dk/services/MLST/; accessed on 16 February 2023).

#### 2.4.3. Data Availability

Sequence data were submitted to the European Nucleotide Archive (ENA) under BioProject accession number PRJEB61067. Each strain was stored with the accession numbers ERS14859644-ERS14859647, and the genomics sequences can be accessed with the accession numbers ERR11179010-ERR11179013.

## 3. Results

### 3.1. Dog Hospital Procedures and Bacterial Analysis

A sample of the mucopurulent discharge of the left nostril was collected during the emergency appointment and immediately transported to the microbiology laboratory. Microbiological analysis of this first sample detected growth of a pure culture on BA medium, being identified as a *K. pneumoniae* (strain 3055). Antimicrobial susceptibility results of this isolate revealed the expression of extended-spectrum β-lactamases (ESBL). Therefore, the clinicians decided to proceed with a second hospitalization in the isolation ward and administer injectable amikacin (by slow intravenous infusion). During the administration of antibiotics, the dog was kept in the isolation ward, where the following care was performed: (i) cleaning both nostrils every eight hours with saline and nostril aspiration; (ii) total restriction to public space (no access to the street); (iii) proper disposal of all organic (feces, urine) and non-organic materials (e.g., gloves); (iv) no external visits; and (v) the biochemical analysis of blood to monitor urea and creatinine every 5 days, in order to control renal function.

Eight days after the start of injectable antibiotic therapy, a new sample was taken from the left nasal cavity of the dog. In this sample, growth on BA and SCAi media was observed, as it involved a recovered *K. pneumoniae* isolate (strain 3089/2, BA and SCAi) and an *S. pseudintermedius* isolate (strain 3089/1, BA). Both strains revealed a multidrug-resistant profile (ESBL Klebsiella pneumoniae and MRSP). Nevertheless, no alteration was made to the clinical protocol: antibiotic therapy with amikacin in the isolation ward was maintained, which lasted 14 days. After 4 days without medication, the third sample was collected from the left nostril, and only MRSP could be found in microbiological samples. Therefore, the dog was discharged without any antimicrobial therapy. New samples of both nostrils (fourth and fifth samples) were taken six weeks after ending antimicrobial therapy, and no growth on SCAi nor on MAC mediums was observed. The polymicrobial flora presented on these samples were compatible with commensal flora, and none of the previous isolates were identified.

### 3.2. Antimicrobial Susceptibility Testing

The antimicrobial profile of the four isolated strains (two *K. pneumoniae* and two *S. pseudintermedius*) is presented in Table 1. All the strains were resistant to more than three antibiotic classes, being classified as MDR.

The *K. pneumoniae* isolates recovered in this study were classified as ESBL since they were resistant to aztreonam, cefotaxime and ceftazidime (Table 1) [23]. The antimicrobial profile of both *K. pneumoniae* strains (3055 and 3089/2) displayed the same antibiotic susceptibility pattern with resistance to penicillin, cephalosporins, monobactam, macrolides, tetracyclines, fluoroquinolones, folate inhibitor, phenicol, nitrofuran and aminoglycosides antibiotic class. Regarding the aminoglycoside class, both *K. pneumoniae* strains only showed resistance to tobramycin and streptomycin (Table 1).

The two *S. pseudintermedius* isolates recovered were resistant to oxacillin, being classified as MRSP. The antimicrobial profile of *S. pseudintermedius* isolates (3089/1 and 3099) revealed antibiotic resistance to penicillin, cephalosporins, aminoglycosides, tetracyclines, fluoroquinolones, lincosamides and folate inhibitor classes. Although both strains demonstrated susceptibility to doxycycline, the diameter of inhibition was near the lower limit of the breakpoint.

### 3.3. WGS and In Silico Genomic Characterization

#### 3.3.1. ESBL *K. pneumoniae* Strains Characterization

Both ESBL *K. pneumoniae* strains presented the same seventeen acquired genes related to aminoglycoside (*aac(6′)-Ib-cr, aph3-Ia*, *strA* and *strB*), 3rd generation cephalosporins (*bla*_CTX-M-15_), fluoroquinolones (*qnrB1*, *qnrB4*, *gyrA*-83I and *parC*-80I), penicillins (*bla*_DHA-1_, *bla*_OXA-1_, *bla*_TEM-1D_ and *bla*_SHV-11_), phenicols (*catB3*), sulfonamides (*sul1* and *sul2*) and trimethoprim resistances (*dfrA14*) (Table 2). No genes mediating resistance to carbapenems, 3rd generation cefalosporins or penicillins combined with beta-lactamase inhibitors, colistin, fosfomycin, tetracyclines, monobactams, nitrofurans and tigecycline were found. Interestingly, the resistance of *K. pneumoniae* to monobactam, tetracycline and nitrofuran was observed, but no associated genes were identified (Appendix B).

In terms of virulence-associated genes, only the siderophore yersiniabactin gene was found on both ESBL *K. pneumoniae* strains (Table 2).

Both ESBL *K. pneumoniae* strains presented the same three types of plasmids: IncFII(K), IncFIB(K) and IncR. These strains also possessed identical MLST, closest cgMLST, capsule locus and serotype O (11, 1509, KL105 and O1/O2v2, respectively; Table 2).

**Figure 3:**
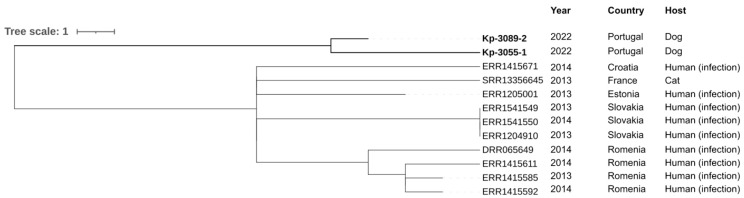
Neighbor-joining tree representing the phylogenetic relationships among *K. pneumoniae* genomes obtained in this study and those available in PathogenWatch with less than 22 SNPs. The cgMLST single linkage clustering was used for genome selection with a threshold of 5 allele differences, and the neighbor-joining tree was inferred from the PathogenWatch pairwise-distance matrix. The metadata of each isolate (country, source and date) was added using iTOL (https://itol.embl.de/; accessed on 10 March 2023).

The neighbor-joining tree generated by comparing the cgMLST of *K. pneumoniae* genomes isolated in this study with those available in PathogenWatch revealed an association with isolates from human infections and a cat (Figure 3). Geographically, this group was identified mainly in Romania and Slovakia, followed by Estonia, France and Croatia (Figure 3).

#### 3.3.2. MRSP Strains Characterization

Genes mediating resistance to penicillins (*bla*Z and *mecA*), macrolides (*erm(B)*), aminoglycosides (*aph(3′)-III* and *ant(6)-Ia*), tetracyclines (*tet(K)* and *tet(M)*), clindamycin (*erm(B))* and trimethoprim (*drfG*) were found on both strains (Table 3). The *aac(6′)-aph(2″*) gene was also detected in the 3099 strain. However, fluoroquinolone, streptogramin or phenicol genes mediating resistance were not found. Resistance of *S. pseudintermedius* to cephalosporin and fluoroquinolones was observed, but no associated genes were identified (Appendix B).

Both isolates have the rep7a and repUS43 plasmid replicons and the Vc(5C2&5) SCC*mec* element. No virulence-associated genes were identified in the MRSP strains (Table 3).

## 4. Discussion

The frequent occurrence of multidrug-resistant bacteria has become a global threat to public health [2]. Overuse of antibiotics has been identified as the leading driver of AMR [30,31]. A case of a dog with rhinorrhea caused by an ESBL *K. pneumoniae* was investigated. Due to the resistance profile presented by this isolate, injectable amikacin was administered, and the animal was hospitalized in the isolation ward. Other causes were ruled out using CT and rhinoscopy. While under antibiotic treatment, ESBL *K. pneumoniae* was again isolated along with MRSP. This last MDR bacterial strain was considered opportunistic [32], derived from the selective pressure and depletion of the natural nasal microbiome caused by the antibiotics [33]. Therefore, no additional treatment was prescribed, and 6 weeks after the antibiotic treatment had ceased, only commensal flora was found in samples from both nostrils.

The antimicrobial resistance profiles of the two strains of *K. pneumoniae* (3055 and 3089/2) showed identical antibiotic susceptibility patterns, both being considered ESBL. The high level of resistance was remarkable, especially for the antibiotic classes of penicillin, cephalosporin and fluoroquinolone, which can be explained by two reasons: these are the most frequently prescribed antibiotics in veterinary medicine [34], and the dog underwent an emergency gastric surgery one week before the first sample collection, in which cephazolin antibiotherapy was prescribed. For instance, in Portugal, fluoroquinolones and cephalosporins represented the second and fourth most often prescribed antibiotic classes in both human and animal medicines [35]. Nonetheless, *K. pneumoniae* is commonly resistant to aminopenicillins [23].

Considering the resistance genes found by PathogenWatch in *K. pneumoniae* isolates, the ESBL phenotype was held by the detection of β-lactamase resistance genes (*bla*_CTX-M-15_; Table 2). Also, fluoroquinolone, phenicol, aminoglycoside, sulfonamide and trimethoprim resistance genes were detected, in accordance with previous studies, which demonstrated that at least 80% of ESBL producers were also resistant to sulfonamides, quinolones and aminoglycosides [36].

Nevertheless, a few discrepancies were found between phenotypic and genotypic resistance profiles. Although *aac(6′)-Ib-cr*, *aph3-Ia*, *strA* and *strB* were detected, ESBL *K. pneumoniae* strains were phenotypically susceptible to gentamycin and amikacin. Likewise, genes mediating resistance to penicillin combined with β-lactamase inhibitors, monobactams, macrolides, tetracyclines and nitrofuran were not found, despite ESBL *K. pneumoniae* strains showing intermediate resistance to tetracycline, doxycycline and resistant to amoxicillin/clavulanic acid, aztreonam, azithromycin and nitrofurantoin.

The virulence gene *Ybt 1* was found in both strains of *K. pneumoniae*, which encode the iron-scavenging siderophore yersiniabactin, promoting systemic survival and dissemination [37]. Also, previous studies demonstrated that this virulence gene favored the maximum growth and lethality of *K. pneumoniae* in respiratory tract infection [38,39], which may have largely contributed to the pathogenicity in this case.

Plasmids often transport resistance genes and virulence genes that can disseminate by horizontal gene transfer mechanisms [40]. IncFII(K), IncFIB(K) and IncR plasmids were detected for both strains of *K. pneumoniae*, being this type of plasmids associated with epidemic *K. pneumoniae* and implicated in the worldwide spread of multidrug resistance [41]. The same authors found an association between the *bla*_CTX-M-15_ gene and the IncR plasmid in ESBL *K. pneumoniae* isolates from Portuguese hospitals [41].

The two strains of *K. pneumoniae* recovered in this study presented the capsular-type KL105 and Sequence Type 11, previously associated with MDR and virulence determinants (yersiniabactin and colibactin) [42]. The ST11 KL105 clade has been successfully disseminated in Europe, even circulating in Portuguese hospitals for years [43,44]. Therefore, it can be hypothesized that a human previously hospitalized or working in a hospital may have had contact with this dog.

Moreover, lipopolysaccharide O locus serotypes were identified as O1/O2v2 for both strains of *K. pneumoniae*, which was associated with hypervirulent strains and was found more frequently in clinical genomes, including in a Portuguese clinical genome [45].

Although strains circulating in Portugal with the same MLST, lipopolysaccharide O locus and capsular type have been described, these Portuguese isolates of *K. pneumoniae* were not available on PathogenWatch, and it was impossible to establish a phylogenetic correlation between them. Therefore, the results of the phylogenetic tree supported kinship (<22 SNPs) to strains mainly from eastern countries (Estonia, Slovakia and Romania) and isolated especially from human infections (Figure 3). These data can be explained by the migration (of both people and animals) from eastern countries to Portugal and the consumption of imported food and feed. In addition, *K. pneumoniae* ST11 was first reported in France (in 1997) and has since been reported all over the world, including in America, Asia and most countries in Europe, such as The Netherlands, Norway, Poland, Slovakia and Portugal [46,47,48]. ST11-*K. pneumoniae* lineage has only been reported in humans, and no data was available in dogs. Moreover, in the 2000s, there was a wave of immigration from Eastern European countries, namely from Ukraine, which is the third country with the largest group of immigrants to Portugal [49]. Also, both strains of *K. pneumoniae* isolated were closely related (5 allele differences).

Since ESBL *K. pneumonia* isolated in this study possessed a myriad of genetic determinants, previously characterized with high pathogenicity and antimicrobial resistance, it was assumed that the isolated strains were at the origin of the mucopurulent rhinorrhea and that veterinary medical action was correctly adjusted to the microbiological findings.

Regarding the results of *S. pseudintermedius* strains (3089/1 and 3099), the isolation of these strains during amikacin treatment may have been caused by antibiotic selection pressure [32]. Hence, the susceptibility profiles were identical. Besides showing resistance to cefoxitin and oxacillin, detection of the *mecA* resistance gene enabled both strains to be classified as MRSP. Similar to ESBL *K. pneumoniae* strains, *S. pseudintermedius* presented resistance to penicillin, aminoglycosides, macrolides, tetracyclines and trimethoprim classes. Moreover, the *aac(6′)-aph(2″)* gene was only detected on the 3099 strain, and it has been described that it confers resistance to a broad spectrum of aminoglycosides [50,51]. The acquisition of another gene to reinforce the resistance to aminoglycosides might have been caused by the selective pressure of amikacin, allowing the bacteria to gain a competitive advantage over other bacteria [52].

However, a few disparities were also found between the phenotypic and genotypic resistance profiles. Indeed, no genes for resistance to fluoroquinolones were identified, and phenotypically, both MRSP strains showed resistance to ciprofloxacin and levofloxacin. These discrepancies observed in the two bacterial species (*K. pneumoniae* and *S. pseudintermedius*) were not pursued. However, potential antimicrobial mechanisms without resistance gene expression include activation of multidrug efflux pumps or decreased outer membrane permeability [53], which should be further explored for fluoroquinolone resistance. Moreover, databases can differ essentially in the number and type of genes and resistance determinants they comprise [54], so there is the possibility that quinolone-resistant determinants were not found due to the database data used. Hence, phenotypic and genomic evaluation are complementary, both being required for a complete account of resistance.

In both strains of MRSP, rep7a and repUS43 were identified. These plasmids frequently carried the *tet(M)* and *tet(K)* resistance genes [55], which is in agreement with antimicrobial resistance genes results.

In addition, the two MRSP strains were identified as ST551, being recorded between 2015 and 2018, 12 *S. pseudintermedius* ST551 strains in the PubMLST database, from different geographical locations and animal hosts (https://pubmlst.org/, last accessed 28 February 2023). Among the 12 records, six samples were isolated from dogs (50%), four from cats (33%) and two from humans (17%). Indeed, *S. pseudintermedius* has been correlated to infections in dogs, being considered an important pathogen in canine pyodermas [56,57]. As for localization, these isolates were from Poland (50%), Switzerland (25%), Sweden (17%) and the USA (8%), evidencing the spread of ST551 throughout Europe since 2015.

Also, both MRSP isolates harbored the SCC*mec* type Vc (5C2&5) element. Since these strains possess the *tet(K)* gene, this is in agreement with previous studies, which showed that isolates carrying the Vc (5C2&5) element co-harbor *tet(K)* in a higher proportion than isolates with other SCC*mec* elements [58].

In this study, the right choice of antibiotic in combination with inpatient hospitalization in the isolation ward might have contributed to the clinical success of the case. While the choice based on the antibiogram allowed the elimination of the primary agent of infection, the isolation of the animal possibly prevented the dissemination and spread of multidrug-resistant bacteria. Although antibiotic therapy may have been at the origin of MRSP recovery, once the inhibitory action of the prescribed aminoglycoside disappeared, the competitive advantage of MRSP on nasal flora dissipated. Thereby, the decision of clinicians to focus only on eliminating the ESBL *K. pneumoniae* strains may have been the correct one.

Some limitations should be considered in the present study. Firstly, data on antimicrobial prescriptions before the emergency appointment were not available. Secondly, since only one clinical case was investigated, some bias in the interpretation of results may be present. Despite these limitations, the results of this study provide valuable information on the dynamics established between the antibiotic and the bacteria during a therapeutic protocol of an infectious process.

Hence, pets can act as reservoirs of AMR genes that may transfer to other inhabitants of the house, both humans and animals. Therefore, veterinary practices, along with microbiology laboratory guidance, must adapt to this new reality, ensuring effective treatment of infections and protection of animal, human and environmental health.

## 5. Conclusions

The present study intended to investigate the effect of antibiotic pressure on the isolation of multidrug-resistant bacteria. Our results showed that antibiotic therapy may have been the cause of antimicrobial resistance and MRSP recovery. The isolation of MRSP followed by its elimination may have been the result of antibiotic pressure for a long period, combined with the competitive action of the commensal flora. The discrepancies observed in this study between phenotypic and genotypic determinants of antimicrobial resistance demonstrated their complementarity. Moreover, the geographical distribution of isolates with similar characteristics to the isolates in this study showed the wide dispersion of the bacteria. Thus, this study highlights the importance of readapting veterinary practices to safeguard the effective treatment of infection and the protection of human, animal and environmental health.

## Figures and Tables

**Table 1:** Antimicrobial susceptibility profile of *K. pneumoniae* and *S. pseudintermedius* isolated from the dog in different sampling times.

Antibiotic Class	Antibiotic	*Klebsiella pneumoniae*		*Staphylococcus pseudintermedius*	
		3055	3089/2	3089/1	3099
Penicillin	AMP	R	R	-	-
	AMC	R	R	-	-
	OXA	-	-	R	R
	PEN	-	-	R	R
Cephalosporins	CAZ	R	R	-	-
	CFZ	R	R	-	-
	CTX	R	R	-	-
	FOX	R	R	R	R
Monobactam	ATM	R	R	-	-
Macrolides	AZM	R	R	R	R
	ERY	-	-	R	R
Aminoglycosides	AMK	S	S	-	-
	GEN	S	S	I	I
	STR	R	R	-	-
	TOB	R	R	R	R
Tetracyclines	DOX	I	I	S	S
	TET	I	I	R	R
Fluoroquinolones	CIP	R	R	R	R
	LEV	R	R	R	R
Ansamycin	RIF	-	-	S	S
Lincosamide	CLI	-	-	R	R
Folate inhibitor	SXT	R	R	R	R
Phenicol	CHL	R	R	S	S
Nitrofuran	NIT	R	R	S	S
Carbapenems	IMP	S	S	-	-
Streptogramins	QDA	-	-	S	S
Oxazolidinones	LZD	-	-	S	S
Sampling order		1st	2nd	2nd	3rd

R, resistant; I, intermediate; S, susceptible; AMK, amikacin, AMC, amoxicillin/clavulanic acid; AMP, ampicillin; ATM, aztreonam; AZM, azithromycin; CAZ, ceftazidime; CHL, chloramphenicol; CIP, ciprofloxacin, CTX, cefotaxime; CLI, clindamycin; DOX, doxycycline; ERY, erythromycin; FOX, cefoxitin; GEN, gentamycin, IMP, imipenem; CFZ: cephazolin; LEV, levofloxacin; LZD, linezolid; NIT, nitrofurantoin; OXA, oxacillin; PEN, penicillin; QDA, quinupristin-dalfopristin; RIF, rifampicin, STR, streptomycin; SXT, sulfamethoxazole/trimethoprim; TET, tetracycline; TOB, tobramycin.

**Table 2:** Whole genome characterization of *Klebsiella pneumoniae*.

Sample ID	Antimicrobial Resistance Genes	Virulence Genes	Plasmid Typing	MLST	Closest cgMLST	Capsule (K) Locus	O Serotype Locus
3055	*aac(6′)-Ib-cr*, *aph3-Ia*, *strA*, *strB*, *bla*_CTX-M-15_, *qnrB1*, *qnrB4*, *gyrA*-83I, *parC*-80I, *bla*_DHA-1_, *bla*_OXA-1_, *bla*_TEM-1D_, *bla*_SHV-11_, *catB3*, *sul1*, *sul2*, *dfrA14*	*ybt 1*	IncFII(K), IncFIB(K), IncR	11	1509	KL105	O1/O2v2
3089/2	*aac(6′)-Ib-cr*, *aph3-Ia*, *strA*, *strB*, *bla*_CTX-M-15_, *qnrB1*, *qnrB4*, *gyrA*-83I, *parC*-80I, *bla*_DHA-1_, *bla*_OXA-1_, *bla*_TEM-1D_, *bla*_SHV-11_, *catB3*, *sul1*, *sul2*, *dfrA14*	*ybt 1*	IncFII(K), IncFIB(K), IncR	11	1509	KL105	O1/O2v2

**Table 3:** Whole genome characterization of Staphylococcus pseudintermedius.

Sample ID	Antimicrobial Resistance Genes	Plasmid	MLST	SCC*mec* Type
3089/1	*aph(3′)-III*, *ant(6)-Ia*, *erm(B)*, *drfG*, *bla*Z, *mecA*, *tet(K)*, *tet(M)*	rep7a, repUS43	551	Vc(5C2&5)
3099	*aac(6′)-aph(2″)*, *aph(3′)-III*, *ant(6)-Ia*, *erm(B)*, *dfrG*, *bla*Z, *mecA*, *tet(K)*, *tet(M)*	rep7a, repUS43	551	Vc(5C2&5)

**Figure A1:**
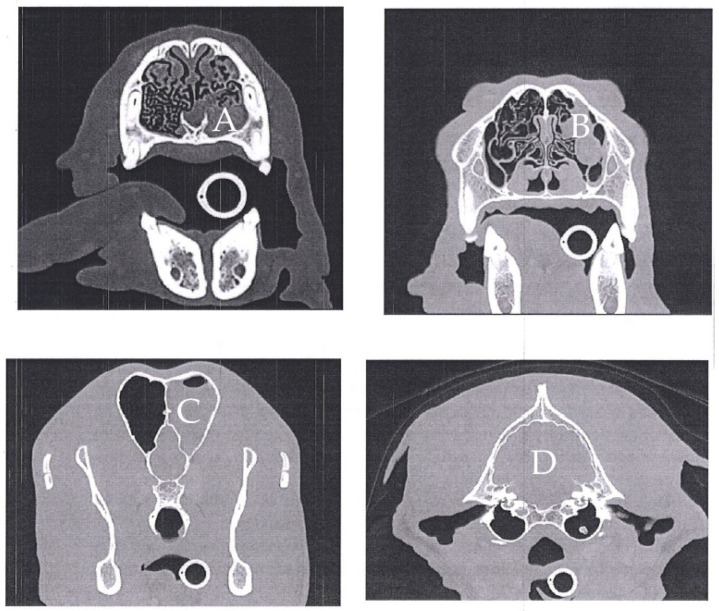
Cranioencephalic computed tomography of a one-year-old Serra da Estrela dog. Left rhinosinusitis with no evidence of a foreign body with the left nasal cavity occupied with a homogeneous material with preservation of the nasal turbinates (**A**). This material extends caudally until the etmoturbinates and left frontal sinus (**B**,**C**), with no signs of osteolysis. The tympanic bullae are regular with normal aerial content, with the exception of the presence of an otolith in the left bulla (**D**). The cerebral parenchyma was normal (**D**).

## Data Availability

Not applicable.

## Appendix B

**Table A1:** Summary of phenotypic resistance and resistance genes by the antibiotic class of the four isolates.

		*Klebsiella pneumoniae*			*Staphylococcus pseudintermedius*		
Antibiotic Class		3055	3089/2	Genome	3089/1	3099	Genome
Penicillin	R	2/2	2/2	*bla*_DHA-1_, *bla*_OXA-1_, *bla*_TEM-1D_, *bla*_SHV-11_	2/2	2/2	*bla*Z, *mecA*
	S	0/2	0/2		0/2	0/2	
Cephalosporin	R	4/4	4/4	*bla*_CTX-M-15_	1/1	1/1	Missing
	S	0/4	0/4		0/1	0/1	
Monobactam	R	1/1	1/1	Missing	na	na	na
	S	0/1	0/1		na	na	
Macrolides	R	1/1	1/1	Missing	2/2	2/2	*erm(B)*
	S	0/1	0/1		0/2	0/2	
Aminoglycosides	R	2/4	2/4	*aac(6′)-Ib-cr*, *aph3-Ia*, *strA*, *strB*	2/2	2/2	*aph(3′)-III*, *ant(6)-Ia)*, *aac(6′)-aph(2″) **
	S	2/4	2/4		0/2	0/2	
Tetracyclines	R	2/2	2/2	Missing	1/2	1/2	*tet(K)*, *tet(M)*
	S	0/2	0/2		1/2	1/2	
Fluoroquinolones	R	2/2	2/2	*qnrB1*, *qnrB4*, *gyrA*-83I, *parC*-80I	2/2	2/2	Missing
	S	0/2	0/2		0/2	0/2	
Ansamycin	R	na	na	na	0/1	0/1	na
	S	na	na		1/1	1/1	
Lincosamide	R	na	na	na	1/1	1/1	*erm(B)*
	S	na	na		0/1	0/1	
Folate inhibitor	R	1/1	1/1	*sul1*, *sul2*, *dfrA14*	1/1	1/1	*drfG*
	S	0/1	0/1		0/1	0/1	
Phenicol	R	1/1	1/1	*catB3*	0/1	0/1	na
	S	0/1	0/1		1/1	1/1	
Nitrofuran	R	1/1	1/1	Missing	0/1	0/1	na
	S	0/1	0/1		1/1	1/1	
Carbapenems	R	0/1	0/1	na	na	na	na
	S	1/1	1/1		na	na	
Streptogramins	R	na	na	na	0/1	0/1	na
	S	na	na		1/1	1/1	
Oxazolidinones	R	na	na	na	0/1	0/1	na
	S	na	na		1/1	1/1	

* *aac(6′)-aph(2″)* was only identified in 3099 strain. All results with intermediate susceptibility were classified as resistant. na, not available.
